# Association of Anabolic Steroid Use With Hypertension and Cardiomyopathy: A Case Study

**DOI:** 10.7759/cureus.71775

**Published:** 2024-10-18

**Authors:** Sarvesh Neupane, Falguni Kalra

**Affiliations:** 1 Internal Medicine, Touro College of Osteopathic Medicine/Montefiore Nyack Hospital, Nyack, USA; 2 Internal Medicine, Sound Physicians/Montefiore Nyack Hospital, Nyack, USA

**Keywords:** anabolic androgenic steroid, anabolic steroid, anabolic steroid abuse, cardiovascular disease, hypertension

## Abstract

Anabolic-androgenic steroid (AAS) use has always been associated with professional athletes and bodybuilders. Many scandals have shed light on the power of anabolic steroids in enhancing human physique and performance, making them ripe for abuse amongst recreational users. However, there are many dangers of anabolic steroid use that have yet to be uncovered or are slowly making their way into becoming general knowledge, especially as it relates to cardiovascular disease (CVD). These risks may remain masked for years before they are discovered, by which time it may be too late to reverse all the detrimental effects. In this article, we report a case of an otherwise healthy 46-year-old Caucasian male with an admitted history of recent anabolic steroid use who arrived in the emergency department with complaints of left lower extremity swelling and pain, spontaneous bruising of the calf, and shortness of breath for two days. After a thorough evaluation, he was discovered to have underlying essential hypertension (HTN) and heart failure with a reduced ejection fraction of 30%. The patient was educated on the likely effects of steroid use on his condition and the ramifications of having uncontrolled HTN and heart failure. He was advised to discontinue steroid use and begin medical management of these conditions. Ultimately, there isn't an established symptom profile for patients using anabolic steroids, and this unique case of a patient with vague symptomatology who was discovered to have underlying congestive heart failure (CHF) and uncontrolled HTN outlines the need for a thorough investigation of cardiac and vascular body systems in this subset of patients.

## Introduction

Cardiovascular conditions were for a long time considered a complication of aging, but we are seeing more reports of heart conditions in younger patients which has broadened the scope of differential diagnosis and led to further investigation as to contributing factors, including the use of anabolic steroids. Anabolic-androgenic steroids (AAS) or anabolic steroids are defined by the National Institute on Drug Abuse as "synthetic substances similar to the male sex hormone testosterone and which promote the growth of skeletal muscle and development of male androgenic sexual characteristics in both men and women" [1]. The history of anabolic steroid use dates back to 1870 when physician-scientist Charles-Édouard Brown-Séquard self-administered an aqueous extract of canine and bovine testes and reported dramatic rejuvenating effects, leading to its widespread public use [2]. It was much later when Adolf Friedrich Johann Butenandt and Leopold Ruzicka were able to win the Nobel Prize in Chemistry for synthesizing testosterone in 1939 [3]. Bodybuilders and athletes were the first to popularize the use of anabolic steroids seeing its potential to improve performance, most notably in 1954 by a Russian weightlifter [3]. It was eventually banned from international competition shortly after the 1968 Olympics [2]. It is still commonly used in competition as people have developed clever ways to evade detection. The World Anti-Doping Agency (WADA) estimates that 1-2% of athlete's urine samples test positive for performance-enhancing drugs [3]. In an anonymous survey of 2,167 world-class amateur athletes, it was found that 43.6% admitted to using performance-enhancing drugs [3]. WADA estimates that around 3.3% of teenagers use steroids in high school [3].

In medicine, anabolic steroids have been in use as early as the 1940s in the management of burns, trauma, and surgery [4]. With increasing research, they have been proven effective when applied in the treatment of a host of other conditions including hypogonadism, osteoporosis, cachexia caused by human immunodeficiency virus (HIV), and cancer [4]. Ongoing research has hinted at its potential in treating leukemia by stimulating bone marrow proliferation and hematopoiesis, by promoting growth in Turner's syndrome, and in the treatment of breast cancer [4]. Other widely recognized effects of testosterone therapy include enhancing sexual function, increasing muscle mass, and improving physical function in aging individuals [4]. Although the literature is limited in establishing a strong causal relationship between anabolic steroids and cardiovascular dysfunction, there are some studies and reports which link steroids to pathways that have been implicated in cardiovascular disease (CVD), and it is something healthcare providers should be aware of in the management of these patients. When anabolic steroid use is suspected or ascertained, it is helpful to perform a thorough cardiac workup on patients regardless of the presenting symptoms or lack thereof. For instance, patients with underlying hypertension (HTN) can live years without symptoms or have vague symptoms and suddenly develop life-altering medical conditions such as acute myocardial infarction (MI) or stroke if their blood pressure is not adequately controlled [5]. People with congestive heart failure (CHF) live many years without symptoms prior to being diagnosed [5].

## Case presentation

History of present illness (HPI)

A 46-year-old Caucasian male with a past medical history significant for asthma and HTN and recent anabolic steroid use presented with a chief complaint of shortness of breath and chest pressure for 2-3 days along with a bruise on his left calf for one week. He reported having a cough for one month. The patient had redness of the sclera of the left eye, no recent travel, and no history of blood clots. A brief review of systems was positive for persistent dry cough without hemoptysis, fever, chills, or chest pain. The patient admitted to diaphoresis, sore throat, abdominal pain, constipation, bruise to the left calf, and pain and redness to the left eye without halos or blurred vision. He denied leg swelling, nausea, jaw pain, or pain with eating, moving, or postural changes. His vision and visual field were intact.

The patient admitted to occasional cigar and alcohol use. He had a past medical history of asthma. The rest of his social and medical history were noncontributory. His vitals were taken and were as follows: blood pressure (BP): 167/107; heart rate (HR): 101; temperature: 98.7°F (37.1°C); (oral) respirations: 18; and oxygen saturation (SpO2): 98% on room air. He was in no acute distress and conversing freely. His respiratory effort was normal and he was not in any distress. No crackles, rhonchi, or rales were heard on auscultation. There was a noticeable end expiratory wheeze at the left lung base. On the cardiovascular exam, he had a tachycardic rate with a soft systolic murmur. He had a healing 1"×3" ecchymosis to the left medial calf that was present on skin examination. The patient was neurologically intact with no focal deficits. 

The patient's D-dimer, troponin, high-density lipoprotein (HDL), low-density lipoprotein (LDL), and thyroid-stimulating hormone (TSH) were within normal range. B-type natriuretic peptide (BNP) was elevated at 271.1 pg/mL (normal range: <100 pg/mL). Hemoglobin was elevated at 17.9 g/dL (normal range: 13.8-17.2 g/dL). LDL was elevated at 110 mg/dL (reference range: <100 mg/dL). Complete blood count (CBC) was within normal limits. The drug screen was negative (see Table 1).

**Table 1:** Patient labs with reference values BNP: B-type natriuretic peptide; LDL: low-density lipoprotein; HDL: high-density lipoprotein; TSH: thyroid-stimulating hormone

Name	Patient's value	Reference value
D-dimer	0.29	<0.4 μ/mL
Troponin	<0.3	0 and 0.04 ng/mL
BNP	217.1	<100 pg/mL
LDL	110	<100 mg/dL
HDL	47	35-65 mg/dL for men, 35-80 mg/dL for women
TSH	3.91	0.5-5 mIU/L
Hemoglobin	17.9	13.8-17.2 g/dL for men, 12.1-15.1 g/dL for women

In the emergency department, the patient's initial presentation was concerning for acute pulmonary embolism given his sudden-onset shortness of breath and chest pressure. Nitroglycerin 2% ointment 1 inch topical and furosemide 20 mg IV were promptly given for acute chest pain. A chest X-ray, electrocardiogram (EKG), and left lower extremity Doppler were ordered, and the patient was admitted to the extended care unit (ECU) for further evaluation and management.

Hospital course

**Figure 1:**
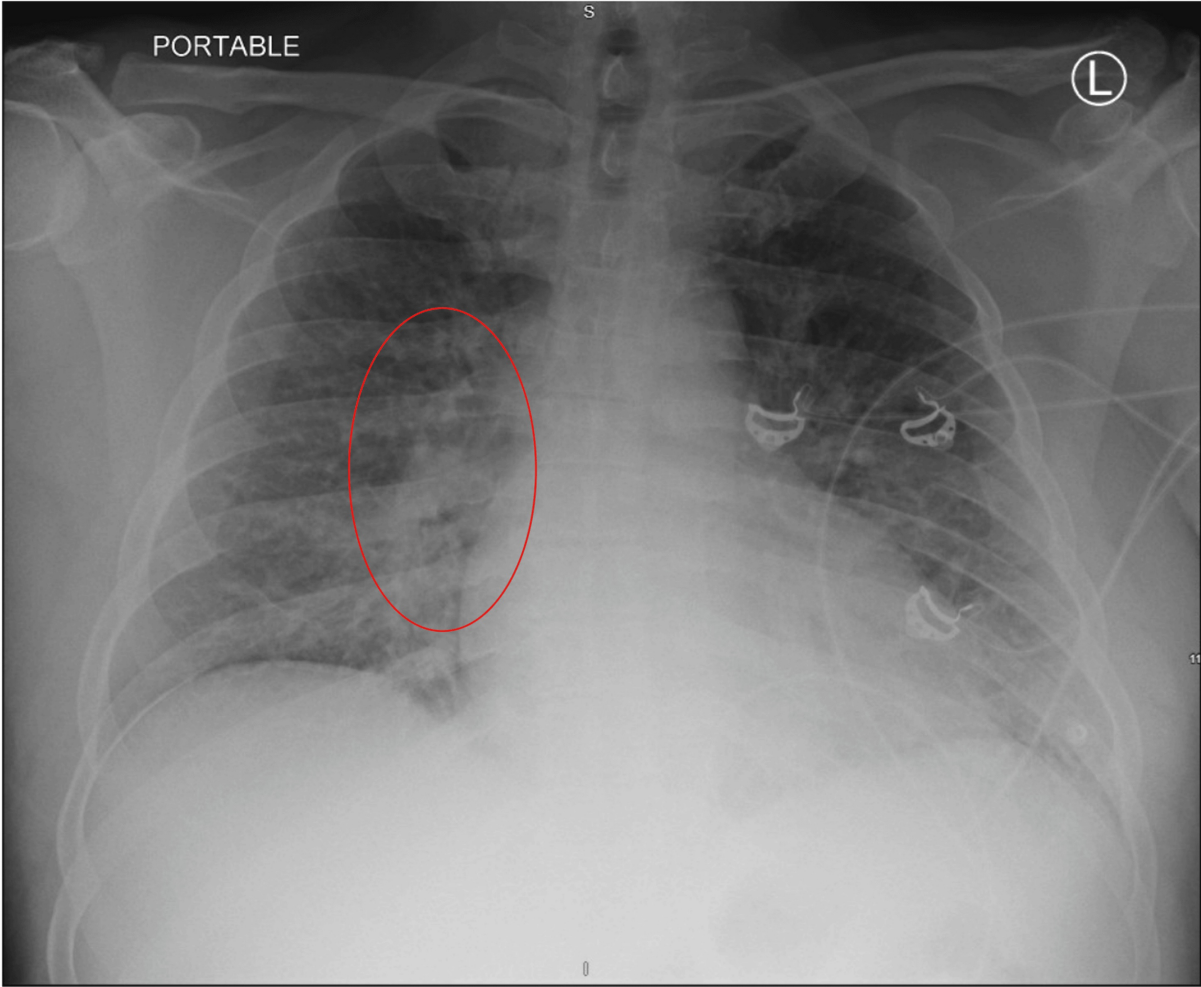
Coronal view of the chest X-ray The red circle depicts one area of diffusely prominent bronchovascular lung markings, possibly related to vascular congestion and/or bilateral pneumonia. Of note, the costophrenic angles are sharp. Hilar structures appear unremarkable. The trachea is midline.

**Figure 2:**
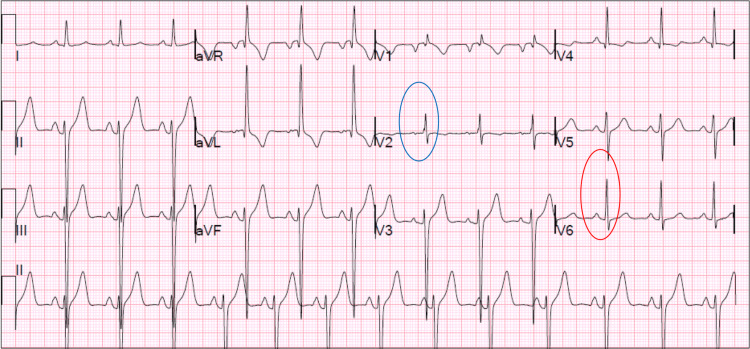
12-lead EKG Sinus rhythm, left ventricular hypertrophy (red circle), left anterior fascicular block, possible right ventricular hypertrophy (blue circle), and anterior T-wave abnormality could be due to hypertrophy and/or ischemia. HR: 80 PR = 166ms QRS = 90 ms QT = 392 ms QTc = 427 ms EKG: electrocardiogram

Following the transfer, the patient was seen in the ECU in the morning. He complained of persistent dry cough without hemoptysis, fever, chills, or chest pain. Cardiology evaluated the congestive changes on the chest radiograph (CXR), and they suspected the patient warranted close observation for CHF versus hypertensive cardiomyopathy in the setting of elevated BNP and HTN, respectively (Figure 1). A sequential compression device (SCD)/Lovenox for deep vein thrombosis (DVT) prophylaxis was given. A 12-lead EKG showed sinus rhythm, left ventricular hypertrophy, left anterior fascicular block, possible right ventricular hypertrophy, and anterior T-wave abnormality that could be due to hypertrophy and/or ischemia (Figure 2). Echo was ordered which showed heart failure with a reduced ejection fraction of 30%, mild tricuspid regurgitation/mitral regurgitation, mildly dilated left atrium, and mild thickening of the anterior mitral leaflet. A right and left heart catheterization was done to rule out atherosclerosis. It showed non-obstructive coronary artery disease with 40% stenosis of proximal left anterior descending (LAD) to mid-LAD coronary artery lesion and normal filling pressures.

An ultrasound of the left lower extremity veins showed no evidence of DVT. There were no filling defects within the pulmonary arterial tree on CT pulmonary angiogram without contrast. There was no evidence of acute pulmonary emboli or other pulmonary pathology. There was an incidental 8 mm solitary pulmonary nodule found on the right lower lobe, and the patient was advised to follow up for a non-contract chest CT in 6-12 months per the Fleischner Society guidelines. The patient was also advised to have a renal ultrasound for outpatient follow-up to rule out renal artery stenosis in the setting of uncontrolled HTN. 

Based on the echocardiogram results, a diagnosis of acute respiratory distress secondary to acute systolic heart failure exacerbation with underlying cardiomyopathy with a reduced ejection fraction of 30% likely secondary to suspected uncontrolled HTN and suspected anabolic steroid use was made. The patient was educated on the side effects of anabolic steroids and advised to discontinue its use. This patient was discharged on albuterol sulfate, carvedilol, dapagliflozin, polyethylene glycol, sacubitril/valsartan, and spironolactone.

## Discussion

Several studies have reported positive effects of anabolic steroids, while others have detailed potential adverse effects. Specifically, there has been much debate about the cardiovascular effects of steroids. Since the exact mechanisms for the effects of steroids on the cardiovascular system are unclear, the side effect profile is broad. There are multiple proposed mechanisms that link steroid use to cardiovascular health. 

Thromboxane A2 (TXA2)

A double-blind, placebo-controlled, randomized, parallel-group study conducted by Ajayi et al. demonstrated that testosterone, a naturally occurring anabolic hormone, regulates the expression of platelet TXA2 receptors in humans [6]. The experimental group received testosterone cypionate 200 mg intramuscular (IM) given twice, two weeks apart, while the control group received a saline placebo. Platelet TXA2 receptor density (Bmax) and dissociation instant (Kd) were measured using an indicator of platelet aggregation response to a TXA2 mimetic, 125I-BOP, in both groups before treatment and at two, four, and eight weeks with treatment with testosterone being associated with increased Bmax with peak effects at four weeks and returning to baseline by eight weeks. The Kd values were unchanged [6]. Platelet TXA2 receptor density was positively correlated (r=0.56, p<0.001, n=32 measurements) with pretreatment (endogenous) plasma testosterone levels (range: 215-883 ng/dL) but not Kd [6]. TXA2 has been widely recognized as a key component in CVD due to its acute and chronic effects in promoting platelet aggregation, vasoconstriction, and proliferation and by the success of low-dose aspirin, a COX inhibitor, in reducing the incidence of cardiovascular events in individuals with high atherosclerotic cardiovascular disease (ASCVD) risk [7]. This increase in blood stasis is a hallmark of thromboembolism leading to stroke and MI, amongst other conditions. 

Dyslipidemia 

Additionally, steroids have been shown to cause an imbalance in lipoproteins, called dyslipidemia. A literary review conducted by Glazer, mostly of prospective cohort studies amongst weightlifters with a self-administered dose of two or more anabolic steroids, showed that anabolic steroids caused a consistent decrease in HDL levels across 15 different studies when taking into account the variable dosing in each study [8]. Percent decreases in HDL value ranged from 39% to 70% in the 15 studies, with the decreases in 11 studies being distributed in a narrow 15% (48-63%) range [8]. The weighted average decrease in HDL level was 52% (range: 39-63%) for the prospective cohort studies and 51% (range: 41-70%) for the cross-sectional studies [8]. In 10 other studies he analyzed, there was a weighted increase in LDL of 36%, all of which were statistically significant (p<0.5) [8].

It has been well documented that LDL cholesterol levels are positively correlated with CVD risk and causality has been established. HDL cholesterol, on the other hand, is inversely correlated with CVD risk although a causal role has not been ascertained [9]. Atherosclerosis in anabolic steroid users has been directly associated with dyslipidemia, with an increase in levels of LDL and a decrease in HDL levels seen in anabolic steroid users [10]. 

Protein synthesis and renin-angiotensin-aldosterone system (RAAS) activation contribute to left ventricular hypertrophy****

Some controlled experiments were able to examine the hearts of anabolic steroid users and non-users using Doppler echocardiography for cardiac magnetic resonance imaging (MRI), showing pathological cardiac hypertrophy with steroid use [4]. Marsh et al. were able to demonstrate that androgen receptors are expressed in cardiac myocytes and that androgens can mediate significant hypertrophy of cardiac muscles through reverse transcriptase polymerase chain reaction (rtPCR) analysis of messenger RNA (mRNA) encoding androgen receptors in myocytes of male and female rats [11]. They exposed neonatal cultured myocytes to 1 μmol testosterone, to 1 μmol dihydrotestosterone, or to vehicle for 48 hours and assessed the rate of [3H]phenylalanine incorporation, a widely used marker for the index of hypertrophy at 37°C. Both androgenic steroids increased protein synthesis significantly (p=0.012) [11]. A confirmation study where an androgen receptor antagonist was used to abolish the hypertrophic response further solidified the role of androgen receptor activation as the source of hypertrophy in these mice [11].

A study by Urhausen et al. took 32 bodybuilders or powerlifters, with 15 athletes who had not taken AAS for 12 months and the other 17 who were currently abusing AAS, to analyze the reversibility of the adverse cardiac effects in these athletes [12]. They found that there was a higher ratio of wall thickness to internal diameter in ex-users and users when compared to non-users consistent with concentric left ventricular hypertrophy, even more than a year after discontinued use of these agents [12]. Additional research has shown that AAS use can disrupt the RAAS leading to left ventricular hypertrophy and fibrosis through elevating the blood pressure, direct action on angiotensin type I receptor on cardiac myocytes, and aldosterone-mediated effects [10]. Despite this increase in muscle mass, research shows that contractility and performance actually decrease due to collagen deposition and increased fibrosis, with molecular studies showing an increase in RAAS-dependent extracellular signal-regulated kinase (ERK1/2) and mammalian target of rapamycin (mTOR) activation system leading to increased fibrosis with no changes in alpha myosin heavy chain (α-MHC) expression which is responsible for contractility. These effects culminated in the remodeling of cell gap junctions, cytoskeleton proteins, and calcium handling, leading to disturbances in coordinated contraction and subsequent pump failure [10].

## Conclusions

A young, relatively healthy male came to the emergency department for shortness of breath and left lower extremity pain and swelling. After gathering a thorough history, it was discovered that he recently used anabolic steroids, and medical testing revealed the patient to have uncontrolled HTN and underlying cardiomyopathy with reduced ejection fraction. In patients with a known anabolic steroid use history, I believe a thorough cardiac workup that includes echo, cardiac catheterization, CT angiogram, and EKG may be considered in order to determine the extent of CVD that may be associated with acute or chronic steroid use and to better assist these patients in managing any underlying conditions. This patient was informed of the side effects of steroid use, advised to discontinue its use, treated for underlying CVD, and discharged on the appropriate medications including albuterol sulfate, carvedilol, dapagliflozin, polyethylene glycol, sacubitril/valsartan, and spironolactone. The patient was advised to follow up with a cardiologist one week after discharge. 
